# Evaluation of Anxiety, Suicidal Risk, Daily Stress, Empathy, Perceived Emotional Intelligence, and Coping Strategies in a Sample of Spanish Undergraduates

**DOI:** 10.3390/ijerph18041418

**Published:** 2021-02-03

**Authors:** Estefanía Solla Montero, Francisco Manuel Morales-Rodríguez

**Affiliations:** 1 Department of Legal Medicine, Toxicology and Psychiatry, Faculty of Medicine, 18016 Granada, Spain; estefaniasolla@correo.ugr.es; 2 Department of Educational and Developmental Psychology, Faculty of Psychology, Campus Universitario de Cartuja, University of Granada, 18071 Granada, Spain

**Keywords:** anxiety, suicidal risk, prosociality, empathy, coping strategies, adolescents

## Abstract

Anxiety disorders and suicide are commonly under-recognized issues that can be a public health problem. Adolescents are among the most affected population groups and studying them can prevent serious health problems. These two elements are related, but at the same time, they can only be understood from a multifactorial point of view, so other related variables such as emotional intelligence, empathy, or coping strategies are key to understand their effect on the population. In this study, a series of methods to measure the variables of interest were applied to a specific group of adolescents to determine their mental health levels, focusing on suicide and anxiety episodes. The results reflected average levels with a tendency to be high in the case of anxiety and nonalarming levels in terms of suicide risk, both parameters presenting more worrying values in women. In turn, the correlation between suicide and anxiety was demonstrated considering the other variables (coping strategies, empathy, emotional intelligence, and prosociality). This research has relevant implications for the diagnosis, orientation, and design of psychoeducational and clinical interventions that contribute to the improvement of their well-being and quality of life.

## 1. Introduction

From the New Educational Model, some of the following can be highlighted: the European Higher Education Area [1], the EuroPsy model for the development of competencies [2] and the Organization for Economic Cooperation and Development [3], the need to develop systemic competencies in future graduates, including inter-and intrapersonal psychological resources such as emotional intelligence, empathy, and the capacity to resolve and face problems and adversity.

Emotions are key constructions related to psychological well-being and life-satisfaction, according to classic theoretical models such as those of Goleman [4], Mayer et al. [5], and Bar On [6] and more recent ones such as Bisquerra et al. [7] somehow, in these models, it is evident that emotional skills can be divided into two poles within a continuum. The positive side includes elements such as productive coping strategies, social skills, prosociality, and empathic attitudes and the negative side that may include symptoms such as anxiety, stress, and depression, which, at the same time, may be considered relevant to assess the level of risk and suicidal ideation in a manner consistent with the localized empirical literature (Lew et al. [8], Zhang et al. [9], and Fonseca-Pedrero et al. [10]).

### 1.1. What Is Anxiety?

Anxiety is defined as a state of mind characterized by extreme restlessness, intense emotion, and high insecurity. Psychophysiologists have long recognized the various roles of anxiety in human behavior and mental illness. It should be recognized that the survival value of anxiety is as important to the human experience as pain. Much of the initial impetus for proving the origin and role of anxiety disorders in mental illness comes from books on psychoanalysis, especially those by Freud and his students, which are based on case histories and personal clinical observations [11].

As far as its classification according to the World Health Organization (WHO) a great group exists where the phobic anxiety disorders are included within which would be the agoraphobia, the social phobias, and other types of specific phobias, such as acrophobia, phobias to animals, claustrophobia, and simple phobia, in addition to the nonspecific phobic anxiety disorders. Outside this classification of anxiety-related phobias, we would find other types of anxiety, including panic disorder, generalized anxiety disorder, depressive and anxiety disorder, and other specific and nonspecific anxiety disorders [12].

In terms of prevalence, anxiety disorders are the most prevalent psychiatric disorders and are associated with high morbidity. Within all the recognized types of anxiety, the one that shows a greater prevalence at worldwide level would be the specific phobia, suffered by more than 10% of the population. Following this one is a disorder of panic with a prevalence of 6%, social phobia with less than 3%, and the disorder of generalized anxiety with a presence of around 2%. In addition, it is remarkable that this type of disorder is much more noticeable in women, sometimes double the number than in men.

The ages of onset of the different disorders are very disparate and depend on the type of disorder we are talking about. To exemplify this, we could point out the case of agoraphobia without panic attacks, which usually appears around the age of 20 years, while specific phobias begin to become noticeable at the age of 7 years [13].

Behavioral interventions: anxiety is understood as a response to a stimulus, while it will subsequently act as a stimulus on responses. The interventions focused on the control of this anxiety centering on the theory of impulse on anxiety. The method of approach is based on the identification of the first signs of anxiety, so that, once recognized, techniques for its reduction and control are put in practice. This type of intervention has not been studied widely, so it is not really known if the positive results are maintained in time; however, these positive results are shown in 50% of the cases.

Cognitive-behavioral interventions: anxiety is understood as a response to threatening situations regardless of whether they represent a real risk to the individual. This would arise because of an equation between the probability of the perceived threat and the capacity to confront it. This type of intervention relies on cognitive restructuring focused on the control of the situation by the individual suffering from anxiety and on the identification of the probability of an occurrence of the event in question that causes these levels of anxiety. A failed identification of this probability of occurrence would lead the patient to present difficulties when facing a problem. The rates of effectiveness of this type of intervention are between 50% and 60% and the improvement is persistent over time.

Cognitive interventions with metacognitive elements: these interventions focus on the fact that when anxiety arises in a patient with a generalized anxiety disorder (GAD), it causes concern in the patient. This preoccupation comes from the hand with the physiological hyperactivation that ends in negative beliefs about the preoccupation and in an attempt to control the same one. This, in turn, makes the fact of wanting to control the worry a new focus of concern and, therefore, higher levels of anxiety and negative evaluations of what is happening are generated. The cognitive and somatic symptoms are interpreted in these cases as a loss of control. These interventions focus on the main technique of being aware of the problem and finding a solution adapted to it, playing with imaginative exposure, and focusing on cognitive restructuring of positive beliefs about worry. As for the effectiveness rates, they are among the highest, presenting an effectiveness of between 75% and 77% [14].

### 1.2. Suicidal Risk

Suicide today is an increasingly serious problem in our society, which implies the need to establish concrete definitions to identify the risk factors, considering that the fundamental causes of suicide come from multiple elements and present great complexity [15]. It is considered one of the leading causes of injury and death worldwide and therefore could be said to be a major public health problem. It is estimated that one death due to suicide occurs worldwide every 40 s, thus, one million people worldwide take their own lives every year. It affects more people in the 10–34 age range and is the second leading cause of death among those ages, while in other population groups, it is in tenth place [16].

Epidemiological studies have shown that men have a higher suicide rate than women at all ages, with a ratio of 5:1. However, if we look at the specific case of women, they make a greater number of attempts in the same proportion, in most cases, through poisoning by pesticides or drugs. Men over the age of 50 have the highest percentage of completed suicides, and these are generally due to hanging or the use of firearms. Worryingly, in recent years, the number of suicides among people in the 15–24 age range has increased, with suicides being more common in lower socioeconomic classes, in people with previous psychiatric treatment, with various personality disorders, and a history of substance abuse, and in people who had previously attempted suicide [15].

The causal effects of suicide vary and can include poverty, absence of work, low self-esteem, psychiatric disorders, death of close people, difficulties in love relationships, age, sexual orientation, and situations that generate great stress [17]. Diverse organizations of importance in the world of health, such as the American Association of Suicidology or the National Suicide Prevention Lifeline include the disorders of anxiety-like factors of key risk for suicide. It has been proven that people who have a positive diagnosis of anxiety disorders have a 10% greater probability of committing suicide or having behaviors related to it. Anxiety disorders have been associated with 12% of all suicide attempts produced in the United States [18].

### 1.3. Other Related Variables: Emotional Intelligence, Empathy/Prosociality, and Coping Strategies for Daily Stress

Emotional intelligence: emotional intelligence and anxiety have shown a strong relationship, which can be defined from a cognitive model or from a mixed model. From the first, the so-called cognitive model, we could define emotional intelligence as a set of skills to identify, understand, use, and regulate emotions, which can be self-perceived or measured through specific tests of ability. According to the second model, the mixed one, emotional intelligence is a set of social, emotional, and personal skills or capabilities that influence the effectiveness when facing environmental demands and pressures. It is clear that stress control, as well as intrapersonal and interpersonal intelligence, are variables that help us predict the magnitude of the anxiety response [19].

Empathy/prosociality: studies linking empathy to anxiety are limited. Empathy could be defined as the tendency to understand and share the other’s emotional state or context. Models studying empathy consider two components: the affective component and the cognitive component. The first, the affective component, refers to the emotions of others and is present from birth. The cognitive component refers to understanding these emotions and taking perspective on them, and they first manifest around 8–10 months of age. It is important to consider the multidimensional nature of anxiety when relating it to other factors such as empathy. The study of how the diverse forms of anxiety affect the affective and cognitive components is interesting at the time of better understanding the clinical pictures and developing new approaches at the time of applying the treatments. The skills related to empathy play a particularly important role in the social development of the individual. Difficulties in the control of emotions put highly empathetic individuals in a vulnerable situation in terms of the development of anxiety disorders and depressive disorders. For these people, sharing emotions with other individuals can generate feelings of maladjustment, such as interpersonal distress and guilt. The result of high levels of affective empathy may be the development of behind turns like social anxiety, which includes difficulties in recognizing, understanding, and interpreting the emotions that are shared with others. In contrast, low levels of cognitive empathy can generate experiences of failed social interactions and, in turn, social anxiety and depression [20].

Coping strategies: coping styles are an important variable that measures an individual’s adaptation to situations where he or she is exposed to great stress. These coping styles have three essential dimensions: the coping method used, the approach to coping, and the activity mobilized in coping. Coping strategies are a group of procedures and efforts, either cognitive or behavioral, that are intended to find a solution to the problem and calming the emotional response, which, in some cases, can be excessive and upsetting. When considering this type of strategy, it is necessary to take into consideration the anticipation and control that the patient has over problematic events, those considered uncontrollable or unpredictable, having the greatest negative effect on health [19]. Coping strategies have often been associated with anxiety problems. Several studies, such as that of Díaz et al. [19], show different coping methods depending on whether the study group is patients with or without anxiety. Patients without anxiety usually prefer active and direct coping strategies related to problem solving, seeking help from society, and positive reassessment. As for anxious patients, they also include positive re-evaluation and problem-solving strategies but to a much lesser degree. They tend to adopt a more passive position, evidencing the strategy of cognitive avoidance, which would be the avoidance behaviors of the problematic situation. They avoid confronting the problem so as not to focus on the specific situation, thus avoiding emotional reactions. Strategies related to asking for help from experts and expressing coping difficulties are also more characteristic of individuals with anxiety disorders. They would be considered behavioral strategies, destined to the advantage of the resources that society offers to the patient. Anxious people also use adaptive strategies just like people who do not present any type of disorder, but this adaptation may not be possible due to a lack of the necessary skills [19].

The adaptation to the university context with the new demands generates stress in the students and makes it necessary to guide them in their process of adaptation and adjustment, so it is necessary to evaluate from the first courses, the score they have in the variables selected for this study.

### 1.4. Aims

To study the levels of anxiety, as well as the risk of suicide in a group of emerging adults and correlations between anxiety, suicide risk, and the psychoeducational variables emotional intelligence, empathy, and coping strategies.

### 1.5. Research Hypothesis

High levels of anxiety and suicide risk are expected to be found in emerging adults and statistically significant correlations between anxiety, suicide risk, and the variables emotional intelligence, empathy, and coping strategies.

## 2. Materials and Methods

### 2.1. Design

This was a cross-sectional, observational, descriptive study. An ex post facto design was employed. For this study, a cross-sectional nonexperimental research design was selected involving convenience sampling of student participants. Participants were asked to complete a series of self-report scales with a Likert response format. The data were analyzed using correlation and multiple regression statistics.

### 2.2. Participants

The total initial sample was 198 students who were undertaking a degree in primary education at the University of Granada. The final sample selected for this study was 154 participants, Spanish undergraduates who gave their consent to participate in the study. Of the 154 students, 55.55% were women and 44.44% were men, mostly single, between 18 and 25 years old with an average age of 20.86 years. Regarding recruitment of the sample, participation was voluntary, i.e., the consecutive recruitment of participants was performed. Convenience sampling was used to recruit participants. The inclusion criteria were as follows: (a) being a university student. The exclusion criteria included: (a) not completing each of the questionnaires provided; (b) not being a full-time student, having recognized part-time student status, and/or having requested a single assessment, and (c) being a student with special educational needs. We worked with an incidental sample balanced by gender. This was carried out using G∗power 3.1 software (version 3.1, Institut für Experimentelle Psychologie, Düsseldorf, Germany). This calculation demonstrated that a sample size of 150 students was needed to provide a confidence interval of 95%, with a power of 95%, assuming a bilateral significance level (α) of 0.05. The present study protocol was approved by the Ethics Committee (Granada, Spain, 1574/CEIH/2020). The participants completed an informed consent form to participate in the study.

### 2.3. Instruments

Several tools have been applied to assess prosociality, empathy, anxiety, coping strategies, perceived stress, and suicide risk.

#### 2.3.1. Suicide Risk

Plutchik’s Suicide Risk Scale, Plutchick [21]: A scale that aims to discriminate individuals with suicidal tendencies from those who are not suicidal. It is constructed with a series of variables that diverse authors have linked to suicide. It presents the power to discern between individuals without suicidal risk from psychiatric patients. It consists of 15 items with dichotomous responses (yes/no) related to previous suicide attempts, with how strong the current auto lithic ideation is, with feelings of depression and hopelessness and with other related aspects. [22] Among the items used are “*Do you have little interest in relating to people?”* or “*Do you see any hope in your future?”.* The internal consistency of the scale was in the initial study of 0.84 and 0.90. Later, an adaptation was made to the Spanish population that reported an internal consistency of 0.90, which was revised years later by Tomás-Sábado et al. [23] affirming an internal consistency of 0.81. This scale has been used in previous studies, such as those carried out by Santana-Campas et al. [24] in a sample of young Mexicans. For the current sample, the scale presents an internal consistency of 0.87.

#### 2.3.2. Anxiety

State-Trait Anxiety Inventory, STAI, Spielberger [25]: Psychological Inventory consisting of 40 self-report items related to anxiety. In this method, the feelings of restlessness, worry, tension, and stress are considered anxiety. The objective of the STAI questionnaire is to measure two independent concepts of anxiety, i.e., anxiety as a state and anxiety as a trait. According to this model, anxiety as a state evaluates passing emotional circumstances, particularized by subjective feelings of apprehension, tension, and nervousness, which are accompanied by physiological changes such as hyperactivity of the autonomic nervous system. Anxiety as a trait indicates an anxious tendency that presents a relative stability that peculiarizes individuals with a predisposition to perceive situations as dangerous. This is not shown directly in the behavior of the individual and is recognized by the frequency at which this individual experiences it. Higher STAI scores would suggest higher levels of anxiety. It is also used to make diagnoses, distinguish between anxiety and depression, and used for research. Within the part anxiety state, examples of the used items are “*I feel calm*” or “*I feel safe*,” whereas in the part anxiety trait, the items, e.g., “*I feel like crying*” or “*I feel rested*” could be mentioned. It is answered by a scale of values where 0 represents the value *almost never* and 3 the value almost always. [26]. This instrument shows adequate psychometric reliability and validity. The scale presents an internal consistency of 0.94 for state anxiety and 0.90 for trait anxiety [27]. It has been applied in several previous studies, such as the study carried out by Macauley et al. [28] in a group of 183 students to measure their anxiety levels in relation to professions related to the health field. The Cronbach’s alpha coefficient for the current sample for the state anxiety subscale was 0.93, while for the trait anxiety subscale, it was 0.89.

#### 2.3.3. Perceived Stress

Perceived Stress Scale, PSS, Cohen [29]: The most widely used measure of perceived stress worldwide is considered a strong predictor of health and illness. It consists of 14 items and measures the global perceived stress experienced in the last 30 days on a 5-point scale (0 = never, 1 = almost never, 2 = occasionally, 3 = often, 4 = very often) presenting a two-dimensional structure, divided into inverse score (negative writing of articles) and noninverse score (positive writing of articles) [30]. Some of those items are “In the last month, how often have you felt that things are going well” or “In the last month, how often have you thought about the things you still have to do.” The internal consistency for this scale for the current sample was 0.87. It had also been applied previously in various studies such as that of Campo-Arias et al. [31] in a sample of 178 women from a university in Colombia with the objective of measuring stress. It can be considered that it allows an evaluation of the daily stress in comparison with other instruments that have been located in the searches made for this work that allows the valuation of vital events like the one of Holmes et al. [22].

#### 2.3.4. Prosociality

Caprara’s Prosociality Scale, Steca [32]: a scale of 16 self-report items with the aim of evaluating individual differences in prosociality in the adult population, proving its measurement capacity through an analysis of the response theory to the items of the studied sample. This scale has been widely tested and its effectiveness in measurement has been demonstrated, as well as the demonstrated high sensitivity of the 16 items. It includes items that comprise help, such as the item “*I try to help others*” and others that comprise empathy, such as the item “*I intensely share my emotions with others.*” These items should be rated on a scale of 1 to 5, with one representing the value *Never/Almost never* and 5 representing the value Always/Almost always. Prosociality can be defined as the set of voluntary actions that one can adopt to help, care for, assist, or comfort others. We are still in a situation of debate between the psychological components and their critical measurement. It has been seen that prosociality derives from complex psychological and developmental processes that involve attention and evaluation mechanisms, moral reasoning, social competence, and self-regulation capacities. [32] The scale presents an internal consistency of 0.84, as measured by Cronbach’s alpha. [33] It has been applied previously in studies such as that of Mieres-Chacaltana et al. [34] in a sample of 245 people in which the relationship between prosociality and subjective well-being was evaluated. For the present sample, the scale presents an internal consistency of 0.87.

#### 2.3.5. Perceived Emotional Intelligence

The Trait Meta-Mood Scale-24 (TMMS-24) [35,36]: This instrument evaluates perceived emotional intelligence, understood as the ability to control feelings and emotions, to discriminate between them and to use that ability to direct one’s own thoughts and actions. The questionnaire has three dimensions: emotional attention, understood as the capacity to feel and adequately express feelings; clarity, interpreted as an optimal understanding of one’s own emotional states; and repair, which alludes to the capacity for optimal control of emotional states. The questionnaire consists of 24 items rated on a Likert scale from one to five points. Cronbach’s alpha for the current simple was 0.86 for emotional attention, 0.79 for clarity, and 0.61 for repair.

#### 2.3.6. Empathy

Test of Cognitive and Affective Empathy, TECA, López Pérez [37]: generic measure of empathy composed of 33 items. It includes the affective and cognitive components of empathy, considering both its positive and negative aspects, thus providing a much more integrated and accurate view of the construct. To evaluate this empathy, both the understanding of others’ points of view and the comprehension of other people’s emotions are considered. In addition, when evaluating affective empathy, both empathic stress and empathic joy are considered. The items are varied, some examples may be “When a friend is sad, I get sad too” or “I find it hard to cry with what happens to others.” These are answered on a Likert scale with values ranging from 1 (totally disagree) to 5 (totally agree). [38]. This instrument presents adequate psychometric properties of reliability and validity and has already been applied in numerous studies (e.g., Estévez et al. [39] involving 1318 individuals who were victims of school violence). The scale presents an internal consistency of 0.86 [40]. The instrument shows satisfactory reliability and validity, and internal consistency for the present sample ranged between 0.78 and 0.87.

#### 2.3.7. Prosocial Trend Measure

Revised prosocial trend measure, PTMR, Carlo [41,42]: this method was born because in previous studies, no distinction was made between the different forms of prosocial behavior, and they were only put into practice as a global construction. This model shows a greater adjustment in comparison with the alternative models. It consists of 21 items and was designed to examine the structure and functions of a multidimensional measure of prosocial behavior. It includes the analysis of the relationships between specific forms of prosocial behavior and sympathy, perspective taking, and global measurement of prosocial behavior. It evaluates six types of prosocial behavior: public (intent to benefit others in the presence of others), emotional (intent to benefit others but under emotionally charged circumstances), emergency (help under crisis situations), altruistic (help without expecting anything in return), anonymous (help without the gaze of anyone), and out of complacency or obedience (help when others request it). The items used are, e.g., “*I prefer to give money without anyone knowing*” or “*When others feel very sad, I usually help them*,” measured by scores from 1 to 5, representing 1 Does not describe me well and 5 Describes me very well [41]. The internal consistency for sympathy is 0.67, for perspective taking is 0.71, and for the overall score is 0.78. [33] This method has been applied previously in studies such as that of Carlo et al. [42] with the objective of measuring prosocial tendencies in a sample of 138 students. This study has shown a Cronbach’s alpha ranging from 0.70 to 0.81.

#### 2.3.8. Coping Strategies

Inventory of Coping Strategies, Tobin (1989), adapted by Cano [43]: After decades of research, this model has proven to be viable in the area of understanding how people handle the stressors in their lives. With a focus on coping strategies, two concepts were developed regarding how individuals manage stressors, namely, coping styles and coping strategies. These, however, are not opposing concepts but complementary. This method was developed from the Coping Modes Scale, and an improvement in this scale has been made. The adaptation is the result of limitations of instruments available in Spanish, modifying the original inventory from 72 items to 40 items. This method collects two types of information, i.e., qualitative information, in which the individual talks about the stressful situation, and quantitative information, where it measures the frequency of use of certain coping strategies and the level of perceived effectiveness in coping, according to a Likert scale. Consequently, both narratives and scores were obtained. The items that constitute the scale are, e.g., “*I blamed myself*” or “*I spent some time alone*.” They are scored on a scale from 0 to 4, with 0 representing the value “*Not at all*” and 4, the value “*Totally*”. It presents a pent factorial structure where the five variables that are evaluated are neuroticism (or degree of emotional instability), extraversion (or degree of sociability and energy), responsibility (understood as the capacity of self-control and self-determination), openness (or range of intellectual curiosity and esthetic sensitivity), and kindness (the capacity to approach or reject others) [43]. This instrument presents adequate psychometric properties of reliability and validity. The scale presents an internal consistency of 0.66 for the cognitive approach coping strategy, 0.66 for the behavioral approach coping strategy, 0.58 for the cognitive avoidance coping strategy, and 0.53 for the behavioral avoidance coping strategy [44]. This method has been previously applied in studies such as that of Rosas-Santiago et al. [45] in a sample of 42 patients with the objective of measuring the coping strategies in the face of a disease, among many other studies. The present study has shown a Cronbach’s alpha ranging from 0.75 to 0.88.

### 2.4. Procedure

Questionnaires were applied online. For this purpose, anonymity, confidentiality of the information, the possibility of leaving the study at any time without giving any kind of explanation, as well as the informed consent of the participants in the study were assured. The average time to complete the questionnaires was approximately 17 min.

Once the data were obtained, a statistical analysis was performed using an ex post facto design. The analysis was carried out using the computerized statistical package SPSS 22.0. An exploration (descriptive statistics and Kolmogorov–Smirnov normality tests) of the study variables and the fulfillment of the assumptions of normality and noncollinearity for Pearson’s correlation were done.

Analyses of the relationships between the quantitative variables were carried out using Pearson’s correlation analysis and linear regression to itself as descriptive analysis and Pearson’s correlation between the variables. Finally, Student’s *t*-test was applied to independent samples to analyze statistically significant differences between students according to gender.

### 2.5. Ethical Aspects

Respect for the dignity of the subjects and the protection of their rights and privacy was prevailed by ensuring that they ticked the consent box before proceeding to fill in the questionnaires.

The project was endorsed by the Research Ethics Committee of the University of Granada (1574/CEIH/2020), which states that the study respects the principles established in international and national legislation in the field of biomedicine, biotechnology, and bioethics as well as all the rights derived from the protection of personal data.

### 2.6. Data Analysis

SPSS software (version 22.0, IBM Corp., Armonk, NY, USA) was used for statistical analysis. First, the descriptive analysis was calculated, and the normal distribution of variables was confirmed using the Kolmogorov–Smirnov test. A scatter diagram was used to verify compliance with the assumptions of linearity and homoscedasticity and determine whether to apply parametric or nonparametric tests. Pearson’s correlation coefficient was used to determine the association between suicide risk and anxiety and each of the psychoeducational variables of everyday stress, prosociality, emotional intelligence, empathy, and prosocial trend measurement.

Normality of residuals, homogeneity of variance for residuals, and linearity of data were examined before completing the regression model. The data met all the assumptions required to carry out the multiple linear regression analyses. Multicollinearity was avoided by selecting a stepwise method in the regression model. A *p* < 0.05 was used as the significance level in the study. Multiple linear regression analyses were conducted to determine the relationships between anxiety and suicide risk, everyday stress, prosociality, perceived emotional intelligence, empathy, and prosocial trend measurement.

## 3. Results

Descriptive Statistics of the Study Variables

For the final sample of participants in this study, 154 who had completed all the questionnaires applied were selected. Of the 154 students, 55.55% were women and 44.44% were men, mostly single, between 18 and 25 years old with an average age of 20.86 years.

Table 1 shows the results of all the tests applied, organized in maximum and minimum scores of each test, and the average of the values obtained from the answers of the participants with their corresponding standard deviation (descriptive statistics).

In general, mean values are presented with a tendency to be high for all measured variables. Among the data that stand out is that of prosociality, whose average value in the population studied is 64.33, a value close to the maximum that allowed the scale to be measured (77.00). As for anxiety, it presents intermediate values in both types of measured anxiety, emphasizing its high standard deviation of the sample, which indicates that the data, in general, do not adjust to the average, consequence of the presence of a great variability among the participating students. However, values with lower levels also stand out, such as the measure of public prosocial tendency, whose average value in the population is 5.89, with a minimum of 3.00 and a maximum of 15.00. In this same case, there are two coping strategies, which are self-criticism with an average value in the population studied of 7.26 (with a minimum of 0.00 and a maximum of 20.00) and the avoidance of problems with a value of 6.17 (with a minimum of 0.00 and a maximum of 16.00).

The results indicate that suicide risk ranged from 0 to 11, with an average of 4.28 (SD 2.45). As for the parameter of suicide risk, the cut-off point for considering a person or a population group being at worrying levels of suicidality is set at 6. This sample is 4.28; therefore, it is important to note that this investigation provided useful resources to subjects who reported scoring above the accepted threshold and a program was designed to follow-up with subjects. Gender differences in anxiety and suicide risk are present.

Table 2 presents the analysis of differences in the anxiety and suicide risk variables in the study according to gender.

In Table 2, we can observe that the variable that presents the greatest difference between genders is suicide risk, with almost twice the risk in the case of women. Regarding anxiety, state anxiety is the one that presents the greatest difference between genders, being 20% higher in women.

The dependent variables (anxiety and suicide risk) exhibited a normal distribution. After the normality of the residuals in the regressions was tested, it was confirmed that the observed residuals were distributed normally. The independence of data for regression was confirmed. There was a linear relationship between the independent and dependent variables. Homogeneity of the residuals’ variance was not violated, and the data met the assumption of homoscedasticity.

Table 3 shows the correlations between the variables of suicidal risk, anxiety, and the other study variables. We found statistically significant associations between suicidal risk and anxiety state, anxiety trait, and everyday stress. We found statistically significant associations between anxiety state, anxiety trait, and emotional understanding.

Next, the correlation between coping strategies for daily stress and the other parameters studied, such as suicidal risk, anxiety, and emotional intelligence, which are especially relevant for providing useful resources to subjects and the design of a training program for effective coping strategies. This is reflected in the table below, again using Pearson’s correlation coefficient (Table 3).

With a significance level of 0.01, a positive correlation was shown between coping strategy, problem solving, empathy, and emotional support. The self-critical coping strategy showed a positive correlation with suicidal risk, state anxiety, trait anxiety, and daily stress. The emotional expression strategy shows a correlation with empathy and emotional support. Desiderative thinking correlates with state anxiety, trait anxiety, and everyday stress. Finally, the social withdrawal strategy shows a positive correlation with the variable measured of daily stress.

With a significance level of 0.05, the emotional expression strategy showed a correlation with prosocial behavior. Desiderative thoughts are correlated with suicidal risk. Finally, social withdrawal also shows a positive correlation with suicidal risk.

As for the values that showed a negative correlation with a significance level of 0.01, emotional expression was negatively related to state anxiety. Cognitive restructuring shows a negative correlation with both types of anxiety and daily stress.

With a significance level of 0.05, a negative correlation was observed between the problem-solving strategy and daily stress, and between the problem avoidance strategy.

Among the situations that students have indicated as causing stress and for which they have employed the abovementioned coping strategies (Table 3) are as follows: the situation generated by COVID-19, the work situation, problems with organization in studies and failing exams, problems with couples, problems with mobility between countries, family issues such as arguments or divorce, health problems, problems with socialization, problems with anxiety control, economic issues, and deaths.

Some examples of the stressful situations brought by the participants in the study are as follows:

“I have always had anxiety, and lately I have been worse, I should have gone to my psychologist to recover a little, but I’ve been avoiding it, and now this situation of being locked up at home and not knowing what’s going to happen with my career, has me very nervous, and I’m thinking all day about studying and eating, and I don’t know how to improve.”

“At one point, I started thinking about the economic crisis that would come after the confinement and how my parents were going to keep the business going in the face of it, and how I was going to continue studying the next year, and I got caught up in a loop of negative thinking and hopelessness.”

Table 4 shows the correlation between anxiety and the independent variables that obtained a significant level of correlation with it.

Table 5 gives the results obtained in regression analyses of significant models of items of the variable “anxiety.” No collinearity between the variables was included in the regression model.

The daily stress and the coping strategy “emotional expression” were significantly related to the dependent variable, predicting 61.2% of the total variance (r^2^ = 0.612, F (2124) = 2.626, *p* <0.001) of the state anxiety levels.

Daily stress and “suicide risk” were significantly related to the dependent variable, predicting 75.3% of the total variance (r^2^ = 0.753, F (124) = 5.217) of trait anxiety levels.

## 4. Discussion

The study has been directed toward a central line consisting of knowing the suicide risk and the levels of anxiety to which students are exposed, in this case, in a specific group of teenagers. It is important to know these levels in first year students who are in the process of adapting to the conditions of the academic context, which may be one of the most stressful for them. [46] To this end, these concepts have been linked to other relevant variables that are considered relevant and the results have been conclusive. It has been shown a clear correlation between diverse variables that were measured in this study, such as the one presented between anxiety of both types (trait and state) with the suicidal risk, which are the two key points of this work, but at the same time, these were found to be related to other variables such as, e.g., suicidal risk clearly correlated with daily stress and this daily stress, in turn, correlated with trait anxiety, for which it can be considered that the study of the diverse variables makes sense because they are elements with a multifactorial dimension.

As for the division of the group by gender, in this work, it has been observed that women are potentially more susceptible to present anxiety and a greater suicidal risk. This data is consistent with other studies such as those by Gao et al. [47], in which it was also stated that more pronounced levels of anxiety were found in the participating women than in men. In the study by Becker et al. [48] provided us with the information that in their work the suicidal risk in women was double that of men, results which we also reached in this study.

The values obtained in terms of suicidal risk do not show a real risk since they would have to exceed the value 6 (our value is 4.28). However, this does not imply that in the population group studied the risk of suicide does not exist; the value provided is an average of all the results and even if it does not exceed is not a low estimate and as a consequence some of the participants did show values that are most worrying with respect to this issue.

As for the study of coping strategies in relation to a problematic situation, these have also shown a correlation with suicidal risk, anxiety, daily stress, and prosociality. By including in the applied tests, a section where the participants could express a stressful situation in which they would use coping strategies, it has been seen that it is necessary to consider the situation generated from COVID-19 when interpreting the results, since these results have probably been altered by reality and may not be the same in a normal situation.

According to a United Nations report (May 2020), the COVID-19 pandemic may cause a global mental health crisis. This extraordinary situation we experience could cause psychological distress to a large part of the population due to the loss of loved ones, economic instability, and isolation.

It is likely that from this event the number and severity of mental disorders of the population will increase, so it is a necessary involvement of governments to solve this public health problem.

The report also highlights that there are some risk groups that could end up more affected by this situation, including isolated children and young people who cannot socialize with others of their age, health workers due to the reality they live with every day, and infected people. The first studies carried out on this subject already show clear signs of an increase in cases of depression and anxiety in several countries [49].

Comparing our results with other similar studies done to university students, it can be observed that the levels of trait anxiety and state anxiety in a study by Caballero-Dominguez et al. [50] in a population group of a university in Colombia are higher than those obtained in this study. Our data show intermediate values in terms of these parameters, while those of the aforementioned study show higher levels (around 40 points).

In terms of suicide risk, the scale was also used in a study by Aradilla-Herrero et al. [51] in a university population of nursing students. That study had a mean according to the Plutchik scale in terms of suicide risk of 3.03, a value lower than that obtained in our sample (4.28).

This work showed diverse results according to the different crossings of established variables that can also be compared with previous studies. In the study already mentioned in this work by Aradilla-Herrero et al. [51], the correlation between emotional intelligence, depression, and suicidal risk was measured. The results showed a significant association between a variable of emotional intelligence and suicidal risk, which, in turn, was related to depression and anxiety. On the other hand, in a study by Singh [52] conducted on Indian university students, a clear relationship was found between some aspects of personality, stressful episodes, and suicidal risk. In this study, correlations were shown between different variables that are relevant to psychological well-being, as in our study, so it can be considered that, in some way, it can reaffirm the idea of the need to approach the subject from a multifactorial point of view, and the study of anxiety or suicide is not recommended without taking into account anything else the evaluation of said variables but considering them in their relationship with other factors or protective or risk variables such as those considered in this work.

Consistent with some previous studies, it is found that everyday academic stressors are one of the risk factors for suicide in young adults [53,54] especially generated in the first years of university where levels of suicidal ideation are higher compared to other courses [55]. Similarly, other previous research [56] also found that among the reasons contributing to student suicide attempts was study-related stress.

The results of this study show the importance of the emotional dimension and the use of effective coping strategies such as emotional expression that affects anxiety levels, which is one of the factors associated with suicidal risk. This is consistent with other research that highlights the importance of predictors of suicidal risk such as impulsivity and coping skills for emotional management and conflict resolution [57].

With respect to gender, this study, as well as other recent research [57,58], shows statistically significant differences in suicide risk factors based on gender, with women scoring higher than men.

The present study argues, as highlighted in other work in our context [59], the importance of knowledge and assessment of suicidal risk factors in young people such as anxiety and stress for the prevention of suicidal behavior by establishing the appropriate support network and providing useful resources to students in this regard.

### Limitations and Future Research

An interesting line of future research would be to check whether young people who present anxiety carry out the suicidal act as their last option in the face of the failure of the effort applied to solve the problem, a situation that led to a feeling of even greater anguish [60].

Consequently, within the clinical perspective, there is much to be researched. It would be interesting to know what the real role of the hypothalamic pituitary adrenal axis (HPA) is and its importance in this process. A greater knowledge of the level of biological processes that cause uncontrolled cases of anxiety or lead to suicidal behavior would be really useful when it comes to solving these public health problems, since more effective pharmacological treatments could be proposed that would put an end to the problem at its root.

From the perspective of psychology, new studies of the effect of anxiety and suicide would be necessary, especially in young people. Based on these, measures should be adopted to improve the well-being of the population, especially in university students where there are fewer studies, despite the fact that adaptation to the conditions of the academic context makes it one of the most relevant areas in the generation of stress and anxiety.

In this study, we did not evaluate physiological responses of the type of palpitations, sweating, tachycardia, or tremors. To include them in future studies would be useful to approach the subject from all possible scopes and to achieve a greater understanding of it.

Our study may be limited by the application of only self-report measures, and there is a need for a longitudinal study to see how scores change in different courses as well as in other degrees and centers. Nevertheless, we consider a representative and adequate sample of students of this degree and course, considering that the priority interest of this exercise is a basic evaluation of the scores obtained in the first courses in which the students present more anxiety and academic stress [61], to guide them, and design intervention programs to face the changes generated in that situation.

The aim of this project is to promote a “program” with psychoeducational methods that contribute to the improvement of the quality of life, psychological well-being, and the prevention of daily stress generated by the academic situations they have to face, promoting the use of more productive and effective coping strategies as protective factors that contribute to reducing these levels of anxiety and suicidal risk. In further research, it would be interesting to extend this with students from other universities by adding other study variables such as sociodemographic data or academic performance.

Sampling does not support the ideal norms of probability in the sense that the participants decided to participate voluntarily in the study and were not selected at random. Consequently, the data shown are only adjustable to university students of the same characteristics, diminishing the capacity of generalization of the results. The data collected from a single institution may have limited generalizability.

## 5. Conclusions

This study is of particular interest to researchers and service providers in the field of student mental health. Adequate mental health is essential at any level of life, especially in adolescence and in the university period (late adolescence), so it is necessary to take it into consideration at the same level as physical health.

This research has shown that suicide risk is associated with emotional skills such as anxiety and daily stress. Anxiety levels were positively correlated with daily stress and coping strategy for emotional expression. The emotional expression strategy shows a correlation with empathy and emotional support. Desiderative thinking was positively correlated with state anxiety, trait anxiety, and everyday stress.

The data obtained will be of use for educational interventions intended to improve psychological well-being and support services with strategies that will enhance students’ management of emotions, prevent stress, suicide risk, and anxiety in the educational setting and lead to better academic performance. Not only this is an interesting paper covering an issue that involves public health but also it seems to be especially important for research and practice given the current worldwide panorama.

## Figures and Tables

**Table 1:** Descriptive statistics of suicidal risk, anxiety, daily stress, prosociality, perceived emotional intelligence, empathy, measures of prosocial tendency, and coping strategies.

Variables	Min	Max	*Mean*	*SD*
**Suicide risk**	0.00	11.00	4.28	2.45
**Anxiety**				
Anxiety State	0.00	55.00	24.88	12.69
Anxiety Trait	5.00	54.00	25.98	11.28
**Daily stress**	24.00	63.00	44.62	9.38
**Prosociality**	28.00	77.00	64.33	8.19
Prosocial behavior	11.00	30.00	24.77	3.45
Empathy and emotional support	7.00	20.00	15.79	2.53
**Perceived Emotional intelligence**				
Emotional care	10.00	39.00	28.30	6.32
Clarity of feeling	14.00	38.00	27.37	5.89
Emotional repair	13.00	40.00	28.40	6.12
**Empathy**				
Adoption of perspectives	19.00	40.00	30.15	3.88
Emotional understanding	25.00	49.00	36.32	5.07
Empathic stress	17.00	40.00	28.21	4.12
Empathic joy	21.00	35.00	29.80	3.58
**Prosocial trend measurement**				
Public	3.00	15.00	5.89	3.01
Emotional	7.00	25.00	18.72	3.77
Emergency	5.00	15.00	11.46	2.06
Anonymous	4.00	20.00	11.65	4.22
Altruist	5.00	20.00	16.99	3.26
Complacency or obedience	4.00	10.00	8.38	1.40
**Coping strategies**				
Problem solving	0.00	20.00	11.68	5.33
Self-criticism	0.00	20.00	7.26	6.49
Emotional expression	0.00	24.00	14.56	6.61
Desiderative thinking	0.00	16.00	11.36	4.58
Cognitive restructuring	1.00	20.00	10.67	5.36
Avoidance of problems	0.00	16.00	6.17	3.52
Social withdrawal	3.00	16.00	10.36	2.49

**Table 2:** Contrast of means (Student *t*-tests) to analyze differences in function of gender in the variables of the study.

Anxiety and Suicide Risk	Man		Woman		Student *t*-Test			
	*M*	*SD*	*M*	*SD*	*t*	*df*	*p*	*d*
Anxiety state	16.17	8.11	24.66	15.62	−1.96	128	0.05	−8.49
Anxiety trait	24.05	9.28	27.60	12.81	−0.90	128	0.07	−3.54
Suicide risk	2.86	1.81	4.34	2.46	−2.31	128	0.02	−1.48

*df*: degrees of freedom; *p*: level of significance; *t*: Student’s *t*-statistician for two independent samples; *d*: difference in averages; *M*: mean; *SD*: standard deviation.

**Table 3:** Correlations between suicidal risk, anxiety, and daily stress, prosociality, perceived emotional intelligence, empathy, and prosocial trend measures.

	1	2	3	4	5	6	7	8	9	10	11	12	13	14	15	16	17	18	19
**1**	1	0.34 **	0.45 **	0.43 **	0.02	0.08	0.04	0.03	0.08	−0.07	−0.08	−0.06	−0.09	−0.05	−0.03	−0.06	−0.01	−0.09	−0.04
**2**	0.34 **	1	0.71 **	−0.00	−0.05	−0.05	−0.08	0.03	0.00	0.12	−0.19 *	0.05	0.05	−0.17	−0.02	−0.05	0.17	0.05	0.00
**3**	0.45 **	0.71 **	1	0.70 **	−0.04	0.09	−0.01	−0.11	−0.12	0.05	−0.28 **	0.12	−0.00	−0.16	−0.02	−0.15	0.16	−0.01	−0.01

* *p* < 0.05; ** *p* < 0.01; 1. Suicidal risk; 2. Anxiety State; 3. Anxiety Trait; 4. Daily stress; 5. Prosocial behavior; 6. Empathy and emotional support: 7. Emotional care; 8. Clarity of feelings: 9. Emotional healing; 10. Adoption of perspectives: 11. Emotional understanding, 12. Empathic stress; 13. Empathic joy; 14. Public prosocial behavior; 15. Emotional prosocial behavior; 16. Prosocial behavior in emergency situations; 17. Anonymous prosocial behavior, 18. Altruistic prosocial behavior, 19. Prosocial behavior out of complacency or obedience.

**Table 4:** Correlations between coping strategies and suicidal risk, anxiety, daily stress, prosociality, and perceived emotional intelligence.

Variables	Problem Solving	Self-Criticism	Emotional Expression	Desiderative Thinking	Cognitive Restructuring	Avoidance of Problems	Social Withdrawal
**Suicide risk**	−0.15	0.31 **	−0.08	0.21 *	−0.13	−0.04	0.21 *
**Anxiety**							
Anxiety State	−0.06	0.36 **	−0.28 **	0.29 **	−0.22 **	−0.08	0.08
Anxiety Trait	−0.07	0.50 **	−0.18	0.34 **	−0.25 **	0.02	0.09
**Daily Stress**	−0.20 *	0.33 **	−0.14	0.34 **	−0.38 **	−0.17	0.20 *
**Prosociality/Empathy**							
Prosocial behavior	0.10	−0.10	0.24 *	−0.04	0.03	−0.08	−0.02
Empathy and emotional support	0.25 **	−0.03	0.33 **	−0.03	0.16	−0.04	0.07
**Emotional Intelligence**							
Emotional care	0.00	−0.15	−0.00	−0.01	0.03	0.02	0.21
Clarity of feeling	−0.08	−0.09	−0.01	−0.03	−0.00	0.01	0.04
Emotional repair	−0.00	−0.04	−0.09	−0.09	0.06	−0.06	0.02

* p < 0.05; ** *p* < 0.01.

**Table 5:** Regression of “anxiety” from the variables of suicidal risk, daily stress, prosociality, perceived emotional intelligence, empathy, measures of prosocial tendency, and coping strategies.

**Anxiety State (r^2^ = 0.612)**						
**Independent Variables**	**B**	**IC 95%**		***β***	***SE***	***p*-Value**
		**Lower Limit**	**Upper Limit**			
Daily stress	0.72	0.37	1.06	0.58	0.17	0.00
Emotional expression (Coping strategy)	−0.49	−0.97	−0.01	−0.27	0.23	0.04
**Anxiety Trait (r^2^ = 0.753)**						
**Independent Variables**	**B**	**CI 95%**		***β***	***SE***	***p*-Value**
		**Lower Limit**	**Upper Limit**			
Daily stress	0.49	0.234	0.743	0.41	0.12	0.00
Suicide risk	1.16	0.193	2.138	0.25	0.48	0.02

r^2^, determination regression coefficient; B, estimators of regression coefficients; *β*, estimators of standardized regression coefficients; CI 95%, 95% confidence interval for B; *β*, adjusted coefficient of multiple linear regression analysis; *SE* coefficient standard error; *p*: level of critical significance.
